# Transdiaphragmatic rupture of a calcified hepatic hydatid cyst into the bronchial tree: CT findings of a bronchobiliary fistula

**DOI:** 10.1016/j.radcr.2026.09.049

**Published:** 2026-10-03

**Authors:** Julien Colafrancesco, Sylvain Grange, Rémi Grange

**Affiliations:** Department of Radiology, University Hospital of Saint-Étienne, Hôpital Nord, Saint-Étienne, France

**Keywords:** Hydatid cyst, Cystic echinococcosis, Bronchobiliary fistula, Transdiaphragmatic rupture, Computed tomography

## Abstract

Transdiaphragmatic rupture of hepatic hydatid cysts into the bronchial tree is a rare but severe complication that may mimic pulmonary infection. We report the case of a 61-year-old man who presented with fever, dyspnea, and right thoracic pain, with initial CT findings suggestive of pneumonia and pleural effusion. Despite antibiotic therapy and surgical drainage, clinical deterioration persisted. Bronchoscopy revealed biliary secretions, and thoracoabdominal CT demonstrated a calcified hepatic hydatid cyst communicating with the right lower lobe bronchial tree through a diaphragmatic defect, establishing the diagnosis of a bronchobiliary fistula. This case highlights the crucial role of thoracoabdominal CT in identifying the hepatic origin of refractory pulmonary disease and in demonstrating the fistulous tract, which is essential for surgical planning.

## Introduction

Hydatid disease (cystic echinococcosis), caused by the larval stage of Echinococcus granulosus, is a worldwide zoonosis that remains endemic in sheep-raising regions, particularly in North Africa, South America, and Eurasia [1]. Humans act as accidental intermediate hosts following ingestion of parasite eggs through contaminated food or close contact with infected dogs [2]. After intestinal absorption, oncospheres enter the portal circulation, explaining the predominance of hepatic involvement, which accounts for approximately 75% of cases, followed by the lung [3]. Hydatid cysts typically grow slowly and may remain clinically silent for years. Imaging plays a central role in diagnosis, allowing lesion characterization, staging, and detection of complications.

Complications are most often the initial mode of presentation and include superinfection, mass effect and rupture. Among these, rupture represents the most serious event and may occur into the biliary tree, peritoneal cavity, or thoracic structures [4]. Transdiaphragmatic extension of hepatic hydatid cysts into the thorax is rare and results from progressive erosion of the diaphragm, facilitated by mechanical pressure, inflammation, and adhesions between the liver and adjacent structures. Rupture into the bronchial tree is an exceptional and severe complication (approximately 3%) [5], leading to the formation of a bronchobiliary fistula [6]. Clinical manifestations may include cough, expectoration of cystic material (hydatidoptysis), or bilioptysis, and are often associated with significant morbidity [7]. Early diagnosis of this entity is critical, as imaging findings may demonstrate diaphragmatic discontinuity, pulmonary consolidation, cavitation, and direct fistulous communication, which are essential for diagnosis and surgical planning. We report a rare presentation of transdiaphragmatic rupture of a hepatic hydatid cyst into the bronchial tree, highlighting key imaging findings.

## Case report

A 61-year-old man, originally from Algeria and living in France for 20 years, was admitted to the emergency ward with a 3-day history of right basithoracic pain associated with dyspnea and fever. His medical history was limited to known opioid dependence treated with methadone; however, history-taking was challenging due to a language barrier. The hepatic hydatid cyst was not known prior to this admission, and no previous investigation or treatment for hydatid disease was documented. On initial clinical examination, crackles were noted over the right lung field and the left lung base. A chest CT scan demonstrated right-sided pneumonia associated with a small pleural effusion. Antibiotic therapy was initiated, and the patient was hospitalized for monitoring. At this time, laboratory tests revealed an inflammatory syndrome without hepatic dysfunction. Despite treatment, the patient experienced progressive respiratory deterioration over the following days. A follow-up CT scan showed a marked increase of right pleural effusion, suggestive of loculated pleurisy. Chest drainage was performed in the operating room, and the patient was transferred to the intensive care unit. Subsequently, he underwent exploratory video-assisted thoracoscopic surgery (VATS) with pleural lavage, which did not result in clinical improvement. Microbiological samples remained negative. A repeat chest CT scan revealed a calcified endobronchial lesion in the right lower lobe, along with a calcified lesion at the hepatic dome on the upper abdominal slices. Blood tests at this time showed a more pronounced inflammatory syndrome, along with newly identified liver function abnormalities, including cytolysis and cholestasis, without associated hyperbilirubinemia. Flexible bronchoscopy was performed but failed to directly visualize the calcified lesion due to limited bronchial caliber. However, it revealed marked inflammation and abundant brownish secretions suggestive of biliary content. A bronchobiliary fistula was suspected, further supported by markedly elevated bilirubin in the bronchial secretions. A subsequent thoracoabdominal CT scan confirmed the presence of a fistulous communication between the calcified cystic hepatic lesion, highly suggestive of a hydatid cyst, and the bronchial tree through the diaphragm (Fig. 1).

**Fig. 1:**
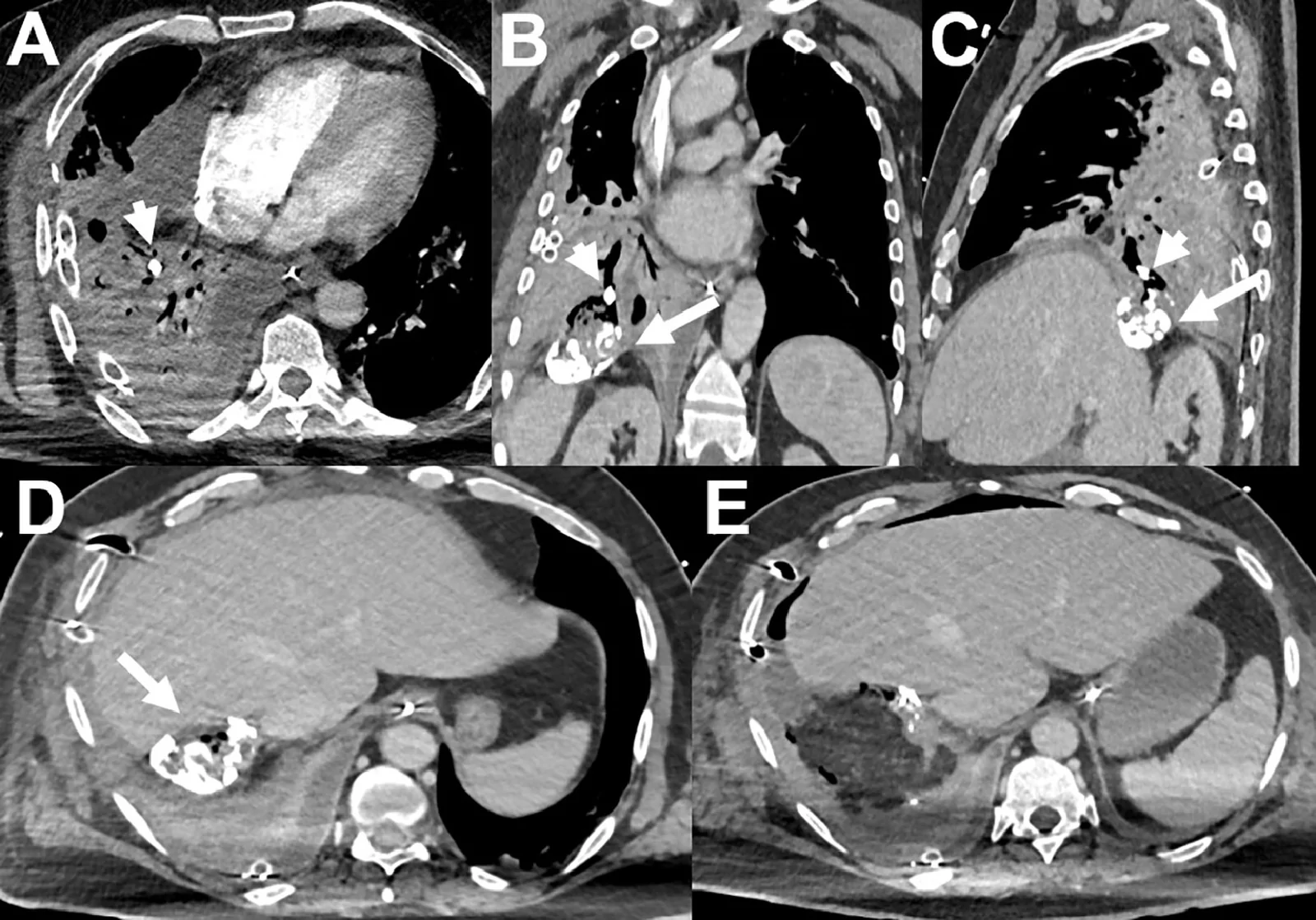
CT findings of transdiaphragmatic rupture of a hepatic hydatid cyst into the bronchial tree. **(A)** Axial chest CT (mediastinal window) showing the calcified hydatid cyst at the hepatic dome with newly appeared intralesional air bubbles, and calcified endobronchial material in the right lower lobe (arrowhead). **(B, C)** Coronal and sagittal CT reconstructions demonstrating the transdiaphragmatic broncho-cystic fistulous tract (long arrow), which crosses a focal defect of the right hemidiaphragm and connects the calcified hepatic cyst, located immediately beneath the diaphragm, to the right lower lobe bronchial tree; calcified endobronchial material migrated into the right lower lobe bronchus is indicated by the arrowhead. **(D)** Axial abdominopelvic CT showing the calcified hydatid cyst with intralesional air bubbles following fistulization (arrow). **(E)** Postoperative axial CT showing the surgical changes after hydatid cyst resection, with no residual cystic lesion.

Following multidisciplinary discussion, the patient underwent surgery consisting of cyst excision, diaphragmatic resection, epiploplasty, and wedge resection of the right lower lobe. Postoperatively, the patient developed an episode of hypereosinophilia, reaching up to 2 G/L. Antiparasitic therapy with albendazole and praziquantel was initiated. The postoperative course was complicated by bilateral pneumonia, intraperitoneal spillage of cyst contents and an epiploic hematoma requiring surgical revision. The patient has since shown significant respiratory improvement and is currently recovering, with planned discharge from the intensive care unit.

Serological testing for echinococcosis was initially negative by Western blot and equivocal by ELISA. However, PCR performed on intraoperative samples ultimately confirmed the diagnosis, which was subsequently validated during a national multidisciplinary team meeting (MDT).

## Discussion

Transdiaphragmatic rupture of a hepatic hydatid cyst into the bronchial tree is an exceptional complication [8]. The pathophysiological mechanism involves progressive erosion of the diaphragm under the combined effect of mechanical pressure exerted by the expanding cyst, chronic perihepatic inflammation, and the formation of dense adhesions between the hepatic dome and adjacent thoracic structures. This gradual process ultimately leads to fistulous communication between the biliary system and the bronchial tree, defining the bronchobiliary fistula. The present case illustrates the diagnostic challenge posed by this entity. The patient initially presented with a clinical and radiological picture mimicking community-acquired pneumonia with parapneumonic effusion, leading to a diagnostic delay of several days. This is a well-recognized pitfall: the thoracic manifestations of transdiaphragmatic rupture — consolidation, pleural effusion, and cavitation — are non-specific and may be indistinguishable from infectious etiologies on initial imaging. Several factors further complicated the diagnostic workup in this case, including the language barrier limiting history-taking, the absence of a previously known hydatid cyst and the atypical presentation without pathognomonic features such as bilioptysis [9] or hydatidoptysis [10]. Although tuberculosis was considered as a possible differential diagnosis given the patient's origin from an endemic country, it was not suspected in our case, in the absence of mediastinal lymphadenopathy or associated pulmonary parenchymal abnormalities; this hypothesis was further ruled out by negative sputum smear microscopy for acid-fast bacilli (AFB).

The key to diagnosis ultimately relied on the convergence of multiple findings: the failure to respond to appropriate antibiotic therapy, the negative microbiological samples, the presence of brownish biliary secretions at bronchoscopy with markedly elevated bilirubin levels, and crucially, the CT demonstration of a calcified cystic lesion at the hepatic dome with direct fistulous communication through the diaphragm to the right lower lobe bronchial tree. This multimodal approach, combining cross-sectional imaging with bronchoscopic findings and biochemical analysis of bronchial secretions, is consistent with the diagnostic strategy recommended in the literature [11]. Of note, bilirubin was markedly elevated in the bronchial secretions while serum bilirubin remained normal, which further underlines the diagnostic value of the biochemical analysis of bronchial fluid when a bronchobiliary fistula is suspected.

From an imaging standpoint, CT remains the cornerstone of diagnosis and surgical planning. The characteristic findings of transdiaphragmatic rupture include: a hepatic cystic lesion with or without calcification or internal membranes, diaphragmatic discontinuity or focal thickening, ipsilateral pulmonary consolidation or cavitation, pleural effusion, and a fistulous tract between the liver and the bronchial tree. In calcified or chronic cysts, as in our case, the parasitic nature may be less immediately apparent, and the calcified endobronchial lesion represented migrated cyst material, a finding that should raise suspicion for this diagnosis when encountered in a patient from an endemic region. The appearance of gas within a previously homogeneous or calcified cyst (the “air-bubble sign”), or an air–fluid level, indicates communication with the bronchial or biliary tree and should not be attributed to superinfection alone. Detached, undulating endocyst membranes within the lesion produce the “water-lily” and “serpent” signs [12], which confirm the parasitic nature of the cyst. The direct demonstration of a fistulous tract crossing a focal diaphragmatic defect and connecting the hepatic cyst to the right lower lobe bronchus is the most specific finding and is best appreciated on thin-section acquisitions with multiplanar and maximum-intensity-projection reconstructions. Multiplanar reformations were decisive in tracing the trajectory of the migrated calcified material from the hepatic dome to the airway and in planning the combined thoraco-abdominal surgical approach. Unlike most reported cases, which present with bilioptysis or hydatidoptysis as the alerting sign, our patient presented with a non-specific refractory pneumonia, making the radiological identification of the hepatic origin the only diagnostic key.

The WHO-IWGE classification of hydatid cysts (CE1–CE5) provides a standardized framework for lesion characterization and guides therapeutic decisions, with CE4 and CE5 corresponding to inactive cysts, CE5 being characterized by a thick calcified wall [13]. In our case, the cyst was classified as CE5, which is uncommon: bronchobiliary fistulization from a fully calcified, inactive CE5 cyst appears to be rarely reported, although complications have still been described with inactive cysts [14]. Treatment of hepatic hydatid cysts complicated by bronchobiliary fistula remains surgical. Conservative or interventional approaches (PAIR: puncture, aspiration, injection, re-aspiration) are contraindicated in the setting of fistula due to the risk of spillage and anaphylaxis. In this case, the surgical procedure, comprising complete cyst excision, diaphragmatic resection, epiploplasty, and wedge resection of the right lower lobe, follows the principles described in the literature, aiming at radical removal of the parasite, closure of the fistula, and management of associated pulmonary and diaphragmatic damage [15]. The postoperative course was marked by significant complications, including bilateral pneumonia and intraperitoneal spillage of cyst contents, underscoring the high morbidity associated with this condition and the importance of early diagnosis before further extension occurs. This case highlights the importance of maintaining a high index of suspicion for transdiaphragmatic hydatid rupture in patients from endemic regions presenting with refractory pulmonary disease. The radiologist plays a pivotal role in recognizing the hepatic origin of thoracic findings and in identifying the fistulous communication, which has direct implications for surgical planning and outcome.

## Conclusion

Transdiaphragmatic rupture of a hepatic hydatid cyst into the bronchial tree is an exceptional and potentially life-threatening complication, which can be misdiagnosed. This case highlights the critical role of thoracoabdominal CT in identifying the hepatic origin of refractory pulmonary disease and the fistulous communication, which is essential for surgical planning.

## Author contributions

All authors contributed to the conception of the work, data acquisition and interpretation, drafting and critical revision of the manuscript, and approved the final version for submission.

## Patient consent

Written informed consent was obtained from the patient for the publication of this case report and the accompanying images.
